# A biopsychosocial approach to primary hypothyroidism: treatment and harms data from a randomized controlled trial

**DOI:** 10.1186/s12998-015-0068-5

**Published:** 2015-08-20

**Authors:** Benjamin T. Brown, Petra L. Graham, Rod Bonello, Henry Pollard

**Affiliations:** Department of Chiropractic, Macquarie University, Balaclava Road, North Ryde, 2109 NSW Australia; Department of Statistics, Macquarie University, Balaclava Road, North Ryde, 2109 NSW Australia; School of Health Professions - Murdoch University, 90 South Street, Murdoch, 6150 WA Australia; Private Practice, 84 Kingsway, Cronulla, 2230 NSW Australia

**Keywords:** Chiropractic, Hypothyroidism, Randomized controlled trial, Therapeutics, Neuro-emotional technique

## Abstract

**Background:**

Hypothyroidism is a common endocrine condition. There is evidence to suggest that, for a proportion of sufferers, the standard medical treatment does not completely reverse the constitutional and neuropsychiatric symptoms brought about by this condition. The management of hypothyroidism follows a biomedical model with little consideration given to alternative management approaches. There exists anecdotal evidence and case reports supporting the use of a biopsychosocial-based intervention called Neuro-Emotional Technique (NET) for this population. The aim of this study was to explore the potential short-medium term clinical efficacy and safety of NET for individuals with primary hypothyroidism.DesignPlacebo-controlled, blinded, parallel groups, randomized trial.

**Methods:**

Ninety adults with a diagnosis of primary hypothyroidism were recruited from Sydney, Australia. Blinded participants were randomized to either the NET or placebo group and received ten intervention sessions over a six week period. The primary outcome involved the measurement of states of depression using the DASS-42 questionnaire. Secondary outcomes included thyroid function, thyroid autoimmunity testing, SF-36v2 questionnaire, resting heart rate and temperature measurement. Outcomes were obtained at baseline, seven weeks and six months. Questionnaires were completed at the private clinics, and serum measures were obtained and analysed at commercial pathology company locations. Heart rate and temperature were also measured daily by participants. Linear mixed-effects models were used to analyse the continuous outcomes. Unadjusted odds ratios with 95% confidence intervals were calculated for the binary outcomes.

**Results:**

Participants were randomly allocated to the NET (n=44) and placebo (n=46) groups. A proportion of the sample displayed neuropsychiatric disturbances and alterations in quality of life measures at baseline. There were no statistically significant or clinically relevant changes in the primary or secondary outcomes between the NET and placebo groups at time seven weeks or six months. There were a few short-lived minor adverse events reported in both the NET and placebo groups that coincided with the application of the intervention.

**Conclusions:**

The application of the NET intervention appears to be safe, but did not confer any clinical benefit to the participants in this study and is unlikely to be of therapeutic use in a hypothyroid population.

**Clinical trials registration number:**

Australian and New Zealand Clinical Trials Registry Number: 12607000040460.

## Background

Hypothyroidism is a common endocrine condition that affects a significant proportion of individuals worldwide. Using data from a community-based sample from an iodine-replete region in Western Australia, it is estimated 0.54 % of Australians suffer from this condition [1]. Females are more likely to develop hypothyroidism compared to males (Prevalence - women 1–2 % and men 0.1 %) [1–3]. The primary variant of this disease accounts for the vast majority (99 %) of cases. Primary hypothyroidism is due to a failure of the thyroid gland and results in a deficient concentration of thyroid hormones in the serum. The most common cause of this glandular failure is an autoimmune condition called Hashimoto’s thyroiditis [4, 5]. The symptoms of overt hypothyroidism are based on deficits in energy metabolism and thermogenesis. Consequently, patients present with a constellation of physical and neuropsychiatric findings (i.e. musculoskeletal complaints, depression, free-floating anxiety, memory deficits) [6, 7], and in cases of thyroid autoimmunity, formation of anti-bodies to thyroglobulin (Tg) and thyroid peroxidase (TPO) [4]. The gold-standard treatment for primary hypothyroidism is relatively straightforward and involves supplementation of one of the deficient hormones using a synthetic version of the thyroid hormone - thyroxine. This synthetic analogue is called levothyroxine (LT4). The treatment dosage of thyroid hormone is gradually titrated upwards until an individual displays normal physiological concentrations of free-thyroxine (FT4) and thyroid stimulating hormone (TSH) in the serum. Put simply, the product of the dysfunctional gland is ‘topped up’ using an exogenous source of thyroid hormone, with the required dose being modified according to the patient’s FT4 and TSH assay results. The patient continues with this program, combined with regular check-ups, either for the rest of their lives or until some event occurs (e.g. pregnancy) that warrants a change in the patient’s thyroxine dosage. The normal reference range for TSH is 0.4–3.5 mIU/L. Patients receiving thyroid hormone replacement are given sufficient quantities of thyroxine to maintain their serum TSH concentrations within an ideal range (0.5–2.0 mIU/L) [8].

While the treatment regimen for primary hypothyroidism is regarded by many as one of the major ‘success stories’ of medicine [9], there is evidence to suggest that a small proportion of individuals with managed-hypothyroidism continue to display the overt manifestations of the disease despite normalised blood test results - indicating a euthyroid state [10–12]. Furthermore, the decreased quality of life (QOL) that is observed in hypothyroid individuals does not always improve with the appropriate treatment [10, 13–15]. Large community-based studies [14, 16, 17] demonstrate that there is neurocognitive and psychological impairment in individuals with hypothyroidism. Unfortunately, in some patients, the restoration of normal thyroid hormone levels through thyroxine supplementation only partially ameliorates these impairments.

One of the main reasons for the persistence of symptoms in euthyroid patients with hypothyroidism is the underlying autoimmune process. Patients with Hashimoto’s thyroiditis are more likely to experience higher symptom loads with reference to mood and quality of life, despite being euthryoid [18]. Furthermore, it is not uncommon for patients with Hashimoto’s thyroiditis to have other comorbid autoimmune conditions e.g. rheumatoid arthritis [19]. The presence of these additional conditions contributes to an increased symptom load that is unaltered by the restoration of normal thyroid hormone concentrations in the serum. McDermott [20] suggests that a complete blood count, serum 25-hydroxyvitamin D, erythyrocyte sedimentation rate, sleep apnoea testing, celiac disease testing as well as a metabolic, hormonal, psychological, and lifestyle assessment should be performed in euthyroid patients with persistent symptoms. This strategy can be used to rule out comorbid medical conditions and/or to identify modifiable risk factors.

Researchers [21, 22] have identified polymorphisms in genes that encode for the deiodinase enzymes in patients with hypothyroidism. There is preliminary evidence to suggest [23] that patients with polymorphisms in the Deiodinase Type II gene may display poorer outcomes on levothyroxine therapy compared with patients receiving preparations containing both levothyroxine and liothyronine (synthetic T3). However, this combination therapy has not proven superior to standard monotherapy [24–27].

With respect to therapeutic intervention, very little attention has been given to the main cause of primary hypothyroidism – thyroid autoimmunity [28]. The main reason for this is that the pathogenesis of thyroid autoimmunity is not fully understood [28]. Current research suggests that autoimmune thyroid disease may result from complex interactions between environmental triggers and genetic susceptibilities [29–32]. The purported environmental triggers include: iodine excess [33, 34], selenium deficiency [35], fetal microchimerism [36, 37], infections [38, 39], and stress [40]. With regard to stress as an environmental trigger, associations have been reported most frequently with Graves’ disease [41–46].

Psychological stress, via alterations in the sympathetic-adrenal-medullary (SAM) axis and the hypothalamic-pituitary-adrenal (HPA) axis, can have deleterious effects on the endocrine and immune systems [47–49]. With respect to the immune system, chronic stress shifts the balance of the cell-mediated immune response, specifically suppressing the subset of T-helper lymphocytes called the Th1 cells [49]. This suppression has a permissive effect on the production of another subset of T-helper cells called Th2 cells [50]. This shift in the Th1/Th2 balance has been observed in certain autoimmune diseases such as Systemic Lupus Erythematosus and Graves’ disease [50, 51]. As a hyperactivation of the HPA axis is a common response to most forms of stress [52], it would be logical to expect a shift to Th2 dominance and a resultant increase in cases of Graves’ disease in genetically susceptible individuals who are stressed. The contribution of stress to Hashimoto’s thyroiditis however is less intuitive. There is some research to suggest that there is HPA axis hypoactivation in patients with hypothyroidism [53, 54]. While hyperactivation of the HPA axis leads to a suppression of Th1 dominant states, a hypoactivation of the HPA axis promotes Th1 dominant states [50]. Autoimmune conditions such as rheumatoid arthritis, Hashimoto’s thyroiditis, multiple sclerosis and diabetes type I are T-cell mediated diseases, however they are thought to occur due to these Th1 dominant states [50, 55, 56]. Hypoactivation of the HPA axis has also been observed in patients with a history of childhood abuse [57] and post-traumatic stress disorder [58], which may be an adaptive response to intense or prolonged psychological stress. The role of stress in Hashimoto’s thyroiditis has received far less attention compared to the role of stress in Graves’ disease [59]. While it is biologically plausible that stress could play an etiological role in genetically susceptible individuals, there are no reported positive associations between stress and autoimmune induced hypothyroidism in the current literature [60–62].

Anecdotal evidence and case reports [63, 64] exist that suggest that a biopsychosocial-based intervention called Neuro-Emotional Technique (NET), which is described as a mind-body stress reduction intervention [65, 66], may be useful in the treatment of hypothyroidism. This technique has been taught and used by practitioners of varying backgrounds since the late 1980s [66], however there is very little quality evidence regarding its therapeutic utility and safety in a primary care setting.

The aim of this manuscript is to outline and discuss the results from a randomized controlled trial of the biopsychosocial-based intervention, NET, for individuals with primary hypothyroidism. This document has been prepared using the CONSORT 2010 guide for the reporting of parallel group randomized trials [67, 68], as well as the associated documents for randomized trials of non-pharmacological treatments [69], and the guide to harms reporting [70].

## Methods

The research presented in this manuscript was approved by the Macquarie University Ethics Review Committee (Reference no.: HE-27AUG2004-MO3136).

### Design

Parallel, blinded, multi-centre (11 sites), randomized, placebo-controlled, trial conducted from August 2006 – March 2010. The study was designed to align with quality recommendations set out in the PEDro scale [71] and The Delphi list [72] as well as with the relevant CONSORT guidelines available at the time of trial design [67, 69, 70, 73, 74].

### Participants

The study team sought to recruit participants from the Sydney Metropolitan area of Australia using advertisements in a variety of hardcopy and electronic media. Participants were randomized into either the NET group or the placebo group after being screened for eligibility by the chief investigator.

### Inclusion criteria - participants

For inclusion, participants had to be ≥18 years of age and have received a diagnosis of primary overt hypothyroidism from a qualified medical practitioner or specialist. In longstanding hypothyroidism there is pituitary thyrotroph hyperplasia [75]. For this reason, it can take up to six months for TSH levels to fall into normal ranges after the initiation of LT4 therapy [76]. Based on this information, if a respondent was taking medication, e.g. levothyroxine, it was stipulated that they be on a stable dose of this medication for at least six months before enrolment into the trial. Acknowledging that treatment is commonly initiated promptly after diagnosis, participants were only included in the trial if they had received their diagnosis more than six months prior to enrolment. Participants were not asked by the research team to alter or cease their thyroid medication at any point during the trial as this would be deemed unethical given the experimental nature of the intervention. However, participants were asked to immediately report any such changes to the chief investigator so that this information could be incorporated into the interpretation of the final results.

### Exclusion criteria – participants

Participants who were <18 years of age were excluded. Individuals with variants of hypothyroidism other than primary overt hypothyroidism, such as hypopituitarism, were excluded. This also included iatrogenic forms of hypothyroidism. Individuals who were on medications that could potentially impair thyroid function e.g. Lithium were excluded. Participants who reported a history of significant head or neck trauma/surgery/radiotherapy were excluded, as were individuals with diagnosed serious psychological or physical co-morbidities. Females who were pregnant or looking to conceive within the trial period were excluded. As participants were required to interact mentally and physically with the practitioner during the intervention phase of the trial, those with cognitive or physical disabilities e.g. individuals who are hard of hearing were excluded from the trial.

### Inclusion criteria – practitioners

It was necessary that the practitioners applying the intervention were well-versed with the NET procedure. Consequently, practitioners were only included if they had attained the maximum qualification available at the time (‘Certification’ in NET) [77] and a minimum experience of two years with the procedure. This ensured a high degree of competency and also acted as a means of standardising the intervention. Practitioners were required to be available for the full duration of the intervention phase for each participant. Practitioners had to agree to treat within the confines of the research methodology and comply with the study protocol, which included the application of a placebo intervention to the relevant participants. The treating practitioners were asked to make times available in their normal practice hours for the treatment of the trial participants. There was no remuneration offered to the practitioners involved in the trial.

### Inclusion criteria – research centres

The research was conducted at 11 private practices. To be included as a research venue, the practice had to fulfil several criteria: be located in the Sydney Metropolitan area, have practitioners with the relevant training and clinical expertise in NET, and be open at least two days per week with capacity for both during and after-hours business operations.

### Intervention

The NET and placebo procedures have been described in depth by several authors [63, 64, 78–84] and it is not within the scope of this article to replicate this discussion. A detailed version of the NET and placebo protocols used in this trial was published in an open-access journal in 2010 [85]. The treatment protocol used in this study was also similar to that employed by Karpouzis *et al.* [78] and Karpouzis [79].

The placebo treatment was developed by a panel that included experts in NET and members of the research team. The placebo intervention was designed to closely mimic the NET intervention protocol but with the purported therapeutic components removed and replaced with innocuous steps.

Participants who were randomized to the intervention group received ten NET treatments over a six week period. Similarly, participants in the placebo group received ten placebo treatments over the same period. Two treatments per week were scheduled in the initial four weeks and one treatment per week in the final two weeks of the intervention phase. There was no further intervention after this point. Each treatment, NET or placebo, went for 5–20 min in duration. Participants attended the same clinic and saw the same practitioner for all of their treatments, all participants attended clinics that were closest to their place of work or residence.

All practitioners involved in the trial were given a training session by the chief investigator, and issued with an information booklet detailing the steps involved with the application of each individual arm of the intervention (NET and Placebo). Practitioners were instructed to deliver the placebo treatment with the same vigour and enthusiasm as the NET treatment. Both practitioners and participants had 24-hour access to the chief investigator at all times during the trial via a toll-free telephone number.

### Outcomes

Several outcome variables were used to capture potential psychological and/or physiological changes that may have coincided with the application of therapy.

### Primary outcome

The primary outcome was the change in depressive states at 7 weeks and 6 months using the Depression, Anxiety, Stress Scale (DASS-42) [86]. The normal range for states of depression is between zero and nine points, with scores greater than nine indicating high states of depression. States of depression was chosen as the primary outcome as it is one of the most common neuropsychiatric symptoms observed in primary overt hypothyroidism, and it is a commonly reported symptom in individuals with continued symptomatology despite normalised thyroid function tests.

### Secondary outcomes

Although it was anticipated that many of the participants would be entering into the trial with pre-existing management plans, i.e. thyroid hormone replacement schedules, it was prudent that thyroid function tests be added to the list of outcomes. The thyroid function tests included the serum measurement of thyroid stimulating hormone (TSH), free-thyroxine (FT4) and free-triiodothyronine (FT3) concentrations. The normal reference range used in this research for TSH was 0.40–3.50 mIU/L and the assay calibration range was 0.00–100.00 mIU/L [87]. The normal reference range used in this research for FT4 and FT3 was 9.0–19.0 pmol/L and 2.6–6.0 pmol/L respectively. The calibration range for the FT3 and FT4 assays was 0.000–9.216 pmol/L and 0.00–77.22 pmol/L respectively [88, 89].

In addition to the thyroid function tests, serum concentrations of antibodies to thyroid peroxidase (TPO-Ab) and thyroglobulin (Tg-Ab) were also measured. The normal reference range for TPO-Ab was 0–35 mIU/L, and the normal reference range for Tg-Ab was 0–40 mIU/L. The calibration range for the TPO-Ab and the TG-Ab assays was 0.0–1000.0 mIU/L and 0.0–3000.0 mIU/L respectively [90, 91].

The remaining items on the DASS-42 questionnaire, states of anxiety and stress, were assessed (Normal Range: Anxiety 0–7, and Stress 0–14). Scores outside of the normal reference range indicate abnormal states.

The Short Form-36 version two (SF-36v2) questionnaire [92] was also included in the list of secondary outcomes. The mean score for a normal population for the domains and component summary scores is 50 norm-based scoring points with a standard deviation of 10 points. High scores on the SF-36v2 represent high functioning whereas scores below the normal mean indicate poorer function.

In addition to the DASS and SF-36v2 questionnaires, participants were instructed to measure their resting heart rate (RHR) (radial pulse method) and resting temperature (RT) at a set time each day and record this data in a diary provided by the researchers. The normal ranges for RHR used for this trial were 75–85 bpm. Participants measured their RT by placing a digital thermometer in their axilla for approximately one minute. The normal reference range for resting temperature used in this research was 35.5–37 °C.

The aim with each of these primary and secondary outcomes measures was to examine the change in each measure at 7 weeks and 6 months to determine whether there was evidence that NET was different (efficacious) compared to placebo. This schedule would allow for the detection of any slow adaptations of the Hypothalamic Pituitary Thyroid axis in response to any short term (7 weeks) and medium term (6 months) benefit derived from the NET intervention.

### Schedule of assessments

Baseline measurements were taken for both the primary and secondary outcomes. Thyroid function tests and thyroid auto-antibody measurements were performed at commercial pathology facilities approximately one week before the commencement of the intervention. The DASS, SF-36v2, and a demographic questionnaire were issued and completed by participants at the respective clinic just prior to the initial treatment. The first follow-up assessment was performed at seven weeks which included; a thyroid function test, serum thyroid auto-antibody measurements, and the DASS and SF-36v2 questionnaires. A second follow-up assessment was performed at six months which was identical to the one performed at seven weeks only with the addition of a health update questionnaire (HUQ) (Appendix). The HUQ was designed by the research team to capture any changes in a participant’s health status, i.e. changes in medication, lifestyle, or major life events in the previous six months that may have influenced the final results and the associated interpretation. The daily home recording of RT and RHR commenced one week before the intervention phase and continued for the full duration of a participant’s enrolment in the trial.

### Blinding survey

The chief investigator contacted each participant by phone after the participant’s final results had been received. The chief investigator thanked the participant for their involvement in the trial and asked them to identify which group, NET or placebo, they felt that they had been allocated to. The participant’s response was recorded and used to assess the adequacy of the blinding process.

### Harms data

A combination of active and passive harms surveillance was used in this study. Practitioners were required to maintain clinical records for each participant visit during the intervention phase. At the beginning of each intervention session participants were asked by the treating practitioner if they had experienced any adverse events in the period that followed the last intervention. Participants were also asked how they felt directly after each intervention. Any responses to these questions were detailed in the participants’ records. In addition to the active harms surveillance participants were also asked to contact the chief investigator if any problems or difficulties arose using the toll free telephone number. These two strategies, combined with information from the HUQ, were used to capture any adverse events that may have coincided with the application of the intervention. These data were compiled and used in the final interpretation of technique safety by the chief investigator. *Adverse events* were defined as an adverse consequence of the intervention (placebo or NET) that occurred any time after the first intervention. Classification of the event as being an ‘adverse event’ was determined by either the participant and/or members of the research team.

### Modifications to the trial protocol

There were several modifications made to the trial protocol between August 2006 and March 2010. In 2007, the pathology company commissioned to analyse the serum measures changed the methodology for analysing the thyroid auto-antibody concentrations. This resulted in the early auto-antibody measures being incompatible/non-comparable to the newer measures. This change prompted the research team to change the TPO-Ab and the Tg-Ab from being continuous variables to dichotomous variables (i.e. either ‘within’ the normal reference range or ‘outside’ of normal reference range) in the final analysis. This resulted in a loss of sensitivity with respect to the minimal clinically important difference detectable for this particular measure.

As the measurement of TSH and FT4 is the standard ‘first-line’ test for individuals with thyroid dysfunction, there were several instances in which the pathology company, due to internal protocols, did not measure the FT3 or thyroid auto-antibody concentrations. Many of these cases were picked up by the chief investigator, however there were instances in which the participant’s sample was destroyed before the chief investigator was able to notify the pathology company of the omission. This resulted in six participants having only partial data for certain variables. This remains a limitation of the study.

As the trial was run over a four year period, there were several changes in the line-up of treating practitioners. Four practitioners started out in 2006. Eight more practitioners were recruited between 2006 and 2010, and four practitioners ceased their involvement in the study during this period due to personal reasons. Despite these changes, each participant received their intervention from a single practitioner.

### Sample size

A two-sided, power-analysis indicated that 51 participants per group would yield a greater than 80 % chance of detecting a difference in depression scores of at least four points in the NET group compared to placebo group using a standard deviation of 6.97 based on normative data [86, 93]. A four point reduction in depression scores, as defined by the DASS-42, would represent a clinically meaningful reduction in states of depression. The ranges for the depression scale are: normal 0–4, mild 5–6, moderate 7–10, severe 11–13 and extremely severe 14+ [86].

### Stopping guidelines

Guidelines were set that dictated that the trial would be stopped in the event of serious adverse events occurring in participants during their participation in the trial.

### Randomization

A simple randomization technique was employed. A random sequence generator [94] was used to produce a random sequence with no repeats. These numbers were then recorded on individual pieces of paper and placed into opaque envelopes which were then sealed. After each consenting participant completed their initial blood test an envelope was selected for that participant by the chief investigator. Participants allocated an even number were assigned to the NET group, and those receiving an odd number were assigned to the placebo group. The chief investigator, practitioners and other researchers remained naive to a participant’s group allocation until just before the application of the intervention.

### Blinding

Pathology company staff and any primary care provider/s involved in the participant’s non-trial management (e.g. the participant’s general practitioner or endocrinologist) were blinded to the group allocations. The allocation of individual participants to treatment groups was concealed to the chief investigator up until the point of a participant commencing treatment. Practitioners were notified of each participant’s group allocation prior to performing the intervention and were instructed not to reveal the allocation to the participant. Individual participants were not told their group allocation until the end of their involvement in the trial and after the completion of the blinding survey (Table 1).

**Table 1:**
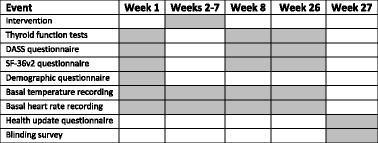
Trial timeline. The trial timeline depicts the timing of all interventions and outcomes measured during the trial

Shaded Area = Event occurrence, *DASS* depression anxiety stress scale, *SF-36v2* short form 36 version 2

### Statistical procedures

The R software program [95] was used to conduct all the analyses. Linear mixed-effects (LME) models were employed for all continuous outcome measures except for the thyroid antibody concentrations. Linear mixed-effects models utilise all available data to provide unbiased estimates of the mean difference between groups in the presence of missing at random data and hence form an intention to treat (ITT) analysis [96]. Since these are more widely used methods, the imputation techniques and statistical methods described in the original protocol were not conducted. The thyroid antibody measures had been dichotomised and logistic mixed-effects models were the planned analysis technique. However model convergence issues meant that it was necessary to calculate unadjusted odds ratios with 95 % confidence intervals for each time point instead. The final data set was screened for errors prior to all analyses. P-values for the interaction and pairwise difference at each time point have been presented for each outcome measure together with 95 % confidence intervals (CI). All participants were analysed according to their initial group allocation. A sensitivity analysis was conducted by adjusting for baseline differences between groups by using baseline data as a covariate in the mixed-effects model. However, the effect of this was that cases with missing baseline data were excluded from the analysis and the analysis may no longer be considered ITT. Demographic characteristics of participants were summarised using frequency tables. Summary values (mean, median, minimum, maximum, and standard deviation) for each group were used for the discussion of baseline group equivalence. Fisher’s exact test was used to analyse the results of the blinding survey. Two-sided tests with a significance level of 5 % were used throughout all analyses. No corrections (e.g. Bonferroni method) were made to the significance level to adjust for multiple testing due to the exploratory nature of the secondary analyses.

## Results

### Post-randomization losses to follow-Up and protocol deviations

**Fig. 1:**
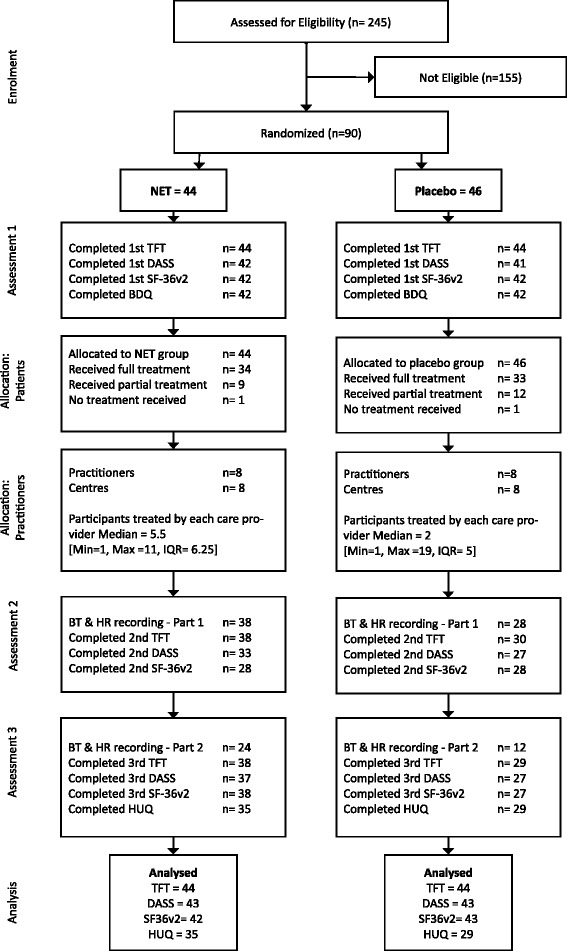
Participant flow diagram. DASS = Depression Anxiety Stress Scale, HUQ = Health Update Questionnaire, NET = Neuro-Emotional Technique, SF-36v2 = Short Form 36 Version 2, TFT = Thyroid Function Test, Min = Minimum, Max = Maximum, IQR = Interquartile Range

The dropouts and protocol deviations that occurred at various phases of the trial are detailed in Fig. 1.

Not all participants in the NET group received the full ten treatments. The reasons for these protocol deviations were either: personal reasons (1/44), administrative error (5/44), the deterioration of a pre-existing medical condition (1/44), minor reaction to treatment (1/44), other time commitments (1/44), and undisclosed reason/s (2/44). With regards to outcome measures, the main reasons for missing data were administrative errors at the private clinics or the pathology company. Several participants in the NET and placebo groups found the daily recording of heart rate and temperature inconvenient and cumbersome. As a result, there was incomplete compliance with the recording of these outcomes.

Not all participants in the placebo group received the full schedule of interventions or completed the full complement of outcomes. The reasons for these deviations were: work issues (1/46), expenses associated with travel to the clinic location (1/46), undisclosed reason/s (5/46), dissatisfaction with the placebo intervention (3/46), other time commitments (1/46), non-compliance with the trial protocol (5/46), and administrative error (4/46).

### Dates of recruitment

Recruitment for the clinical trial took place between August 2006 and March 2010. Two hundred and forty five individuals responded to the advertisements for participation. Eligible consenting participants were followed for six months after randomization.

### Trial closure

A strict external schedule (stopping rule) was imposed that meant that the trial must be commenced and completed between August 2006 and October 2010 due to the length of the chief investigator’s PhD candidature. No interim analyses were performed and hence the decision to close the trial was independent of the trial outcomes. The prescribed schedule meant that no further participants could be recruited after March 2010 despite needing additional participants to fulfil the sample size target.

### Baseline participant data

The demographic information and the mean baseline clinical characteristics for participants are presented in Tables 2 and 3 respectively. The two groups were very similar with respect to demographic data. A comparison of the baseline clinical data is presented below.

**Table 2:** Demographic information

		Treatment (*n* = 44)	Placebo (*n* = 46)
Age	Median	46	43
	Minimum	20	20
	Maximum	72	75
	Mean	45.55	44.61
	Standard Deviation	11.21	11.32
Number of children	Mean	1.51	1.81
	Standard Deviation	1.30	1.20
Gender	Female	43	40
Ethnicity	Caucasian	40	38
	Asian	2	2
	Arab	1	0
	Aboriginal/Torres Strait Islander	0	1
	Unknown	1	5
Birthplace	Australia	26	29
Highest level of education	School Certificate	5	0
	HSC	3	10
	TAFE	13	8
	Undergraduate	13	15
	Postgraduate	8	11
	Unknown	2	2
Marital status	Married	20	27
	Separated	6	5
	Single	14	8
	Widowed	1	0
	Defacto	2	3
	Unknown	1	3
Employment	Full-Time	17	16
	Part-Time	11	13
	Retired	13	11
	Not Working	2	4
	Unknown	1	2

*HSC* higher school certificate, *TAFE* technical and further education

**Table 3:** Mean baseline clinical characteristics

Outcome measure		Treatment		Placebo	
	Normal range	Median (Min, Max)	Mean (SD)	Median (Min, Max)	Mean (SD)
Thyroid function tests:					
TSH (mIU/L)	0.40–3.50	0.96 (0.00, 55.77)	2.98 (8.39)^a^	1.34 (0.01, 7.30)	1.85 (1.76)
FT4 (pmol/L)	9.0–19.0	15.00 (7.00, 26.00)	15.09 (3.22)	14.20 (3.80, 24.50)	14.51 (3.91
FT3 (pmol/L)	2.6–6.0	4.06 (2.80, 7.45)	4.13 (0.80)	3.80 (3.00, 5.73)	3.95 (0.64)
DASS Scores:					
Depression	0–9	6.50 (0.00, 29.00)	9.43 (8.60)	6.00 (0.00, 41.00)	7.85 (8.52)
Anxiety	0–7	6.00 (0.00, 19.00)	6.79 (5.13)	4.00 (0.00, 30.00)	6.27 (7.73)
Stress	0–14	13.00 (3.00, 35.00)	13.71 (8.28)	11.00 (2.00, 38.00)	12.76 (8.53)
SF-36v2 Scores:	Normal Mean (SD)	Median (Min, Max)	Mean (SD)	Median (Min, Max)	Mean (SD)
Physical functioning	50 ± 3 (10)	52.82 (25.46, 57.03)	48.06 (8.36)	50.72 (29.68, 57.03)	48.66 (7.72)
Role physical	50 ± 3 (10)	49.51 (22.57, 56.85)	48.85 (8.60)	51.96 (17.67, 56.85)	46.32 (11.25)
Bodily pain	50 ± 3 (10)	46.06 (24.08, 62.12)	49.97 (9.87)	46.06 (24.08, 62.12)	46.53 (9.43)
General health	50 ± 3 (10)	45.31 (23.38, 57.70)	43.72 (11.26)	45.78 (23.78, 62.47)	45.16 (9.71)
Vitality	50 ± 3 (10)	39.60 (23.99, 58.33)	40.15 (8.88)	41.16 (20.87, 58.33)	41.68 (10.33)
Social functioning	50 ± 3 (10)	45.94 (13.22, 56.86)	42.09 (12.49)	45.94 (13.22, 56.85)	41.12 (12.82)
Role emotional	50 ± 3 (10)	48.10 (20.89, 55.88)	44.78 (10.71)	44.22 (20.89, 55.88)	43.58 (10.96)
Mental health	50 ± 3 (10)	44.38 (21.85, 61.27)	43.79 (11.04)	47.19 (16.22, 58.46)	43.03 (11.62)
Physical component summary	50 ± 3 (10)	50.46 (30.08, 61.73)	48.27 (7.76)	51.39 (16.70, 59.78)	48.45 (9.54)
Mental component summary	50 ± 3 (10)	42.59 (16.70, 60.18)	41.10 (11.49)	41.59 (16.40, 61.35)	40.79 (12.71)
	Normal Range	Median (Min, Max)	Mean (SD)	Median (Min, Max)	Mean (SD)
Resting temperature (°C)	35.5–37.03	35.8 (32.8, 37.0)	35.6 (0.9)	35.6 (33.4, 36.6)	35.6 (0.7)
Resting heart rate (bpm)	75–85	64 (30, 86)	65.7 (9.6)	64 (44, 86)	63.9 (11.3)

The SF-36v2 scores are based on the norm based scoring system (NBS) which is based on a mean score with a normal range between 47 and 53 NBS points. Resting temperature and resting heart rate data were analysed based on ‘Day 1’ dairy entries

^a^Strongly influenced by one person with very high TSH

*DASS* Depression, Anxiety, Stress Score, *NET* Neuro-Emotional Technique, *TSH* Serum thyroid stimulating hormone, *FT4* Free thyroxine, *FT3* Free triiodothyronine, *SF-36v2* Short Form-36 Version 2, *Min* minimum, *Max* Maximum, *SD* standard deviation

### Primary outcome - baseline

**Fig. 2:**
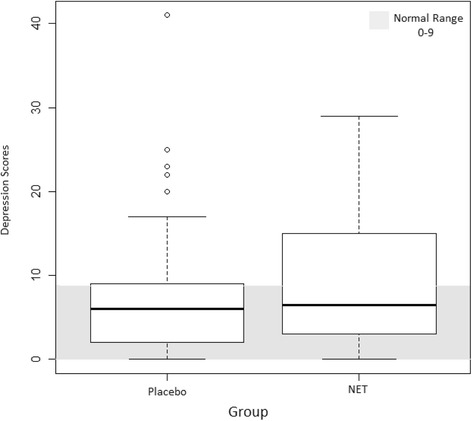
Baseline depression scores

Boxplots of baseline depression scores are depicted in Fig. 2. The mean and median depression scores for the placebo group were within normal range, however the mean score for the NET group fell just outside of the normal range. A large proportion (19/44) of participants in the NET group and 15/46 participants in the placebo group reported states of depression that fell outside of the normal range at the time of initial testing.

### Secondary outcomes and clinical data - baseline

With respect to baseline clinical data, the mean TSH scores for the NET group were skewed by one individual with an extremely high TSH value that remained high at the subsequent seven week and six month reassessments. With reference to thyroid autoimmunity, 30/44 participants in the NET group demonstrated higher than the normal concentrations of TPO-Ab and 12/44 demonstrated higher than normal concentrations of Tg-Ab. Similar results were seen in the placebo group with 30/46 and 14/46 of participants demonstrating abnormally high TPO-Ab and Tg-Ab concentrations respectively.

With respect to the additional DASS items, 17/44 of the NET group and 10/44 of the placebo group reported anxiety scores in the mild to severe range. A considerable proportion of the trial participants also reported mild to extremely severe stress scores (NET 19/44, Placebo 13/44).

The mean baseline scores for the SF-36v2 questionnaire revealed that the trial participants had lower mean self-reported general health (NET 11/44, Placebo 9/46), vitality (NET = 21/44, Placebo 23/46) social functioning (NET 9/44, Placebo 11/46) and mental health (NET 8/44, Placebo 10/46) compared to normative data.

Resting temperature was comparable and within normal range at baseline for both the NET and placebo groups. The data regarding heart rate highlight that the majority of participants in both the NET and placebo groups had lower than normal heart rate compared to the normal range (NET = 37/44, Placebo 44/46).

In addition to the primary and secondary outcomes researchers also gathered data on the previous use of psychological services and concurrent use of medications and/or nutritional supplements. Thirty four percent (15/44) of the NET group and 37 % (17/46) of the placebo group reported that they had used support services (e.g. counselling service) in the past for emotional, psychological or lifestyle problems. At baseline, the vast majority of participants (NET 40/44, Placebo 43/46) reported currently using some form of thyroid medication for the treatment of their condition. With respect to additional medication use, 21/44 of participants in the NET group and 20/46 of participants the placebo group reported using medications other than those prescribed for their thyroid condition. A substantial proportion of trial participants (NET 28/44, Placebo 27/46) reported taking nutritional products or supplements.

### Analysis

**Fig. 3:**
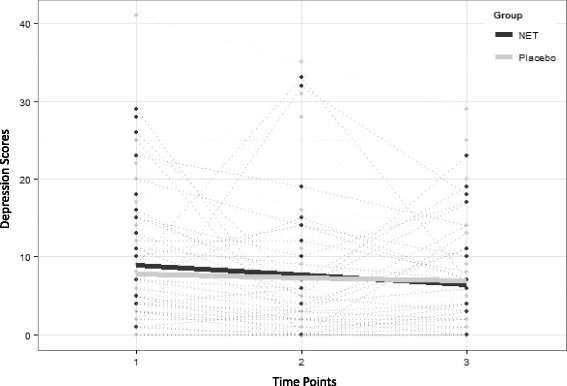
Depression scores over time

The results from the linear mixed-effects modelling for the primary outcome may be seen in Table 4 and Fig. 3. Secondary outcomes that comprised of continuous data are presented in the remaining rows of Table 4. There was no significant interaction between time and treatment group for any of the outcome variables included in the study (final column of Table 4) indicating that there was no evidence of a difference between groups over time (Fig. 3 Primary outcome). There were no significant differences, at the 5 % significance level, between the NET and placebo groups at time seven weeks or six months (see the 3rd and 6th columns of Table 4) for any of the primary or secondary outcomes.

**Table 4:** Analysis of primary and secondary outcomes

Outcome	MCID (±)	Difference between groups 7 weeks (NET-Placebo)		Difference between groups 6 months (NET-Placebo)		Time x group interaction
		Mean difference (95 % CI)	P-value	Mean difference (95 % CI)	P-value	P-value
Depression	4	−0.58 (−4.62, 3.45)	0.78	−0.04 (−4.04, 3.96)	0.98	0.60
Anxiety	5	0.63 (−2.19, 3.46)	0.66	0.36 (−2.44, 3.16)	0.80	0.98
Stress	4	0.29 (−3.63, 4.21)	0.88	1.47 (−2.42, 5.35)	0.46	0.86
TSH	0.75	0.11 (−2.64, 2.88)	0.93	1.01 (−1.76, 3.77)	0.47	0.49
FT4	6.0	0.67 (−0.81, 2.15)	0.37	0.33 (−1.14, 1.80)	0.66	0.85
FT3	1.5	0.29 (−0.24, 0.83)	0.28	0.15 (−0.38, 0.68)	0.58	0.82
Physical functioning	3.5	−0.76 (−9.07, 7.56)	0.86	1.75 (−6.41, 9.91)	0.67	0.35
Role physical	3.2	4.25 (−7.31, 15.81)	0.47	4.35 (−6.93, 15.63)	0.45	0.99
Bodily pain	4.5	3.56 (−7.62, 14.73)	0.53	2.35 (−8.43, 13.13)	0.67	0.76
General health	5.7	2.11 (−8.20, 12.43)	0.69	3.35 (−6.78, 13.47)	0.52	0.28
Vitality	5.5	0.27 (−9.76, 10.30)	0.96	−0.10 (−9.84, 9.65)	0.98	0.76
Social functioning	5.0	9.05 (−4.55, 22.66)	0.19	5.58 (−7.54, 18.69)	0.40	0.65
Role emotional	3.8	2.12 (−8.72, 12.96)	0.70	1.13 (−9.36, 11.61)	0.83	0.86
Mental health	5.5	4.73 (−5.23, 14.72)	0.35	4.83 (−4.84, 14.49)	0.33	0.65
Mental component summary	3.1	4.11 (−1.58, 9.79)	0.16	0.22 (−5.25, 5.69)	0.94	0.36
Physical component summary	3.8	1.02 (−3.14, 5.18)	0.63	0.97 (−3.10, 5.03)	0.64	0.50

*MCID* minimal clinically important difference, *TSH* thyroid stimulating hormone (mIU/L), *FT4* free thyroxine (pmol/L), *FT3* Free triiodothyronine (pmol/L)

There were no significant differences between the NET and placebo groups for either RHR (*p* = 0.20) and RT (*p* = 0.18) readings over time.

The odds ratios and 95 % confidence intervals indicated no significant differences, at the 5 % significance level, between groups in the odds of demonstrating Tg-Ab concentrations outside of reference range at the seven weeks (OR: 0.65, 95 % CI: 0.24 to 1.73, *p* = 0.38) or the six month (OR: 1.24, 95 % CI: 0.45 to 3.38, *p* = 0.68) time points. Similar outcomes were observed with TPO-Ab concentrations (7 week OR: 0.94, 95 % CI: 0.32 to 2.74, *p* = 0.90; 6 month OR: 0.69, 95 % CI: 0.25 to 1.92, *p* = 0.48).

Sensitivity analysis was undertaken by using baseline outcome as a covariate in each of the continuous outcome models shown in Table 4. While the baseline value was consistently a highly significant factor (*p* < 0.0001), the interaction between time and group remained non-significant for all outcomes (*p* > 0.3). In other words, the only differences between groups, were those differences observed at baseline with no significant change over time.

### Harms/adverse events

The harms/adverse events section has been written in accordance with the CONSORT document for the better reporting of harms in randomized trials [70]. No validated harms recording instruments were used.

### Adverse events - NET group

Six (14 %) participants in the NET group reported adverse events that coincided with the application of intervention. One participant in the NET group reported feeling “sad” after the fifth treatment session. This same participant also described feeling “light-headed” after the sixth treatment session. One participant described feeling “drained” after the ninth treatment session. One participant described the sensation of her “head spinning” after the first treatment session. This same participant reported “pain in the lower neck” after the second session and reported a “light headed” feeling after treatments three, four, six and nine. On treatment number nine the participant reported “Always feeling a bit light headed”. On visits seven and eight this same participant reported feeling “tired”. One participant in the NET group developed a sore throat during the middle of the treatment phase which abated after two weeks. One participant reported feeling “a bit sad” after the ninth treatment session. One participant reported feeling “depressed” after the first and second treatment sessions. During the second treatment session, the participant felt uncomfortable while discussing a memory from her past that was prompted by the questioning associated with the intervention. The participant reported that a couple of hours after this treatment that she experienced a migraine headache that lasted for several hours. The participant was unhappy with the nature of treatment and withdrew herself from the trial. The vast majority of adverse events observed in the NET group occurred directly after the application of the intervention and were not reported at the time of subsequent interventions indicating that these events were of a limited duration. The participants who did experience adverse events did not require any treatment aside from reassurance from the associated practitioner. No adverse events were reported in the NET group during the post-intervention phase of the clinical trial.

### Adverse events - placebo group

Six (13 %) participants reported adverse events that coincided with the application of the placebo intervention. Five of the six described feeling “tired” directly after one or more of the placebo intervention sessions. One participant described feeling “sore” after the tapping applied by the treating practitioner to the right inferior angle of the scapula. No adverse events or harms were reported in the placebo group during or after the post-intervention phase of the clinical trial.

### Blinding survey

Of the total number of participants that undertook the blinding survey (*n* = 81), 10/41 (24 %) of placebo participants correctly assumed that they were allocated to the placebo group. Two out of 40 (5 %) participants in the NET group correctly assumed their assignment to the NET group. There was a statistically significant difference between these two proportions (Fisher’s exact test, *p* = 0.025). Based on these results, participants in the placebo group appeared more likely to correctly guess their group allocation compared to individuals in the NET group.

## Discussion

This is the first randomized controlled trial investigating the short-medium term effects of NET treatment on individuals with primary hypothyroidism. Both treatment effects and harms data were captured in this study with the major findings presented below.

The NET and placebo groups were comparable with respect to clinical and demographic data at baseline. The vast majority of the study population were taking some form of thyroid medication, most commonly LT4, for their condition. The mean and median serum TSH, FT3, and FT4 concentrations were within the normal range. The majority of participants displayed abnormal TPO-Ab concentrations at baseline indicating the presence of thyroid autoimmunity. The mean and median depression scores were within normal range however, a substantial proportion of participants displayed abnormal depression scores at baseline. A similar scenario was observed with respect to the anxiety and stress scores at the time of the initial assessment. For the SF-36v2 data, mean self-reported general health, vitality, social functioning, and mental health scores were lower in the study population compared to normative data. These baseline neuropsychiatric findings were expected given the continued symptomatology reported large community based samples of patients with treated hypothyroidism [14, 16, 23].

The results of the analyses revealed that there were no significant differences, at the 5 % significance level, between the NET and placebo groups at time seven weeks or six months for any of the trial outcomes.

If NET has a stress-reducing effect, either; this has not been demonstrated in this particular study; or stress may not be a salient factor in onset and/or progression of this chronic disease. Further research, with much larger sample sizes, is required to ascertain the exact role of stress in the pathogenesis and progression of primary hypothyroidism.

With respect to harms, the way in which the NET treatment was applied, including the dosage, was well-tolerated by the study participants. There were some minor, short-lived adverse-events that followed the application of both the NET and placebo interventions.

### Strengths of the study

This is the first RCT investigating the short-medium term treatment effects of NET in participants with primary hypothyroidism. Practitioners of varying backgrounds with similar clinical expertise in NET applied the intervention to the cohort. The approach to delivery, as well as the dosage of the intervention was based on a private practice model of care. The typical participant in the trial was a Caucasian female with a mean age of 45 years, with evidence of thyroid autoimmunity. This profile aligns strongly with epidemiological data regarding patients with primary hypothyroidism [3, 8, 97]. Due to the systemic influence of the thyroid hormones a wide selection of outcome measures were employed that would have captured any short-medium term physiological and psychological changes that coincided with the application of the intervention.

### Limitations

Designing placebo interventions can be difficult in research when the underlying biological mechanism behind the therapy is unknown [98]. For this reason, designing a placebo in complementary and alternative medicine research is often dependent on unsubstantiated assumptions about the specificity of the intervention [99]. With this in mind, the placebo was designed via the collaboration of a group of NET certified practitioners, and members of the research team. The aim was to create a placebo intervention that would: closely mimic the NET intervention; would not provide any therapeutic benefit; would be considered credible by the participants; and would be of comparable duration to the NET procedure. The results from the analysis of the blinding survey results would suggest that the placebo intervention was more likely to be viewed as a ‘sham’ procedure compared to the NET treatment. As the practitioners were involved in the administration of both the NET and placebo interventions, it was not possible to blind these individuals to group allocation. However, to reduce the problem of non-blinding, practitioners were under strict instruction not to reveal group allocation to participants during the trial, and also to refrain from discussing any of the procedures in detail. Furthermore, as the treatment being provided by the practitioners in their private clinics was not recorded or monitored it was not possible to guarantee the fidelity of either of the treatment arms. Slight differences in the delivery of the NET and placebo intervention may account for the results observed in the blinding survey.

The participant’s subjective report regarding reactions or adverse events associated with the intervention had the potential to be selectively-filtered by the treating practitioners. The majority of practitioners involved in the trial regularly use NET in their private clinics. These practitioners would presumably want to demonstrate the efficacy and safety of the intervention which may have introduced performance and/or reporting bias.

The study was designed to explore the influence of a biopsychosocial-based treatment approach on the clinical manifestations of primary hypothyroidism. The approach involved a diverse number of outcome measures, and multi-centre format in order to make use of all available resources. This scenario can be problematic in that it introduces the statistical problem of multiple comparisons in which the chances of obtaining a false positive result are increased [100]. While no corrections for multiple testing were used given the exploratory nature of the secondary outcomes this type of correction would not have changed the results obtained in this study.

A power analysis indicated that 51 participants per group would yield a more than 80 % chance of detecting a difference in depression scores of at least four points in NET group compared to the placebo group using a standard deviation of 6.97 based on normative data. The fact that the recruitment of participants fell short of the desired sample size means that there is reduced power to find a difference between groups. If we examine how many participants in each group achieved a four point decrease in depression scores at 7 weeks (11/22 for NET vs 7/18 for placebo) then a 95 % confidence interval for the number needed to treat before a positive treatment outcome occurred would be (−19 to 29). This wide interval indicates anything from no benefit to benefit for 1 in 29 participants and reflects the uncertainty in the data collected.

The sample that was obtained aligns well with profiles from large population studies regarding patients with primary hypothyroidism. There is however the potential that a specific type of participant responded to the recruitment advertisements. As the advertisements were calling for participation in research investigating a new, drug-free treatment for hypothyroidism, it is possible that individuals who were either: not taking medication; opposed to the concept of taking medication; poorly compliant with their current medication regimens; or dissatisfied with their current management regimens were more likely to respond. Furthermore, the advertisements made reference to the Department of Chiropractic at Macquarie University, as the host of this research. Proponents of chiropractic/complementary and alternative medicine, or individuals with positive past experiences with chiropractic treatment may therefore have been over represented in the sample population.

Both the DASS-42 and the SF-36v2 have recall periods of less than one month. For this reason, treatment effects with long latency periods may have been missed in between the seven-week and six-month assessments.

Dr Scott Walker [101], the creator of NET, originally designed the technique to be an adjunct to the usual manual therapy procedures he was using in his private practice. Walker deemed psychosomatic stress to be an important factor contributing to continued or cyclical symptomatology in his patients and designed NET to tackle this problem. The technique represented an additional tool that a practitioner could use if the standard manual procedures alone were not having a longstanding therapeutic effect in patients with musculoskeletal conditions [102]. Later on in the development of NET, Walker added nutritional elements and aspects of homeopathy to the NET procedure. In an attempt to make the investigation of NET more manageable, and the results from the study more comprehensible, the research team restricted the intervention to only the component of NET that deals with psychosocial stress. Based on reviews of homeopathy trials, the magnitude and plausibility of treatment effects of homeopathy for specific medical conditions remains poorly defined [103–105]. In a recent report from the National Health and Medical Research Council of Australia a working committee concluded that there were no health conditions for which there is reliable evidence that homeopathy is effective [106]. In the latest guidelines for the management of hypothyroidism, produced by the American Thyroid Association Taskforce, strong recommendations were made against the use of dietary supplements and nutraceuticals [12]. The main reasons for these recommendations were A) the lack of evidence regarding the efficacy of these products and B) the potential risks associated with the use of these products for individuals with thyroid conditions. There is a dearth of high quality evidence regarding manual therapy for the treatment of hypothyroidism and a limited evidence-base for homeopathy and nutrition in this role. It is therefore difficult to ascertain what effect removing these components may have had on the overall efficacy of the NET procedure.

As stress is a potential environmental risk factor for the development of primary hypothyroidism, it is possible that NET treatment, a purported stress reduction technique, may function better in a preventative role. In cases of long standing autoimmune thyroid disease there is inflammation and subsequent destruction of the thyroid follicles. This results in the replacement of the thyroid parenchymal tissue with non-functional fibrous tissue [107]. In this scenario, the pathophysiological consequences of autoimmune thyroid disease have resulted in tissue that no longer has an endocrine function. While the disease process and the associated symptomatology continues, the damaged tissue is unlikely to respond, from an endocrine perspective, to any therapeutic intervention. To determine the preventative effect of NET treatment would require a large scale preventative trial which currently lacks evidential support and is beyond the scope of this discussion.

In obtaining ethics approval for this study researchers were required to outline the foreseeable risks associated with participation in the study. At the time of the trial design there were no published harms data regarding NET. As a result, an information and consent form was drafted that included likely potential adverse events such as muscle soreness, tiredness, and an uncomfortable recall of past events. These events were suggested as potential outcomes during consultation with experts in NET. While there were some minor adverse events that coincided with the intervention in both the NET and placebo groups, there is the possibility that participants were primed [108] to report certain events due to the content of this information and consent form.

## Conclusions

In this study, NET intervention was not found to confer any clinical benefit to the participants in the short or medium term.

The data recorded on harms suggest that there are some minor, short-term, adverse events associated with the application of this procedure. These events however were predictable, relatively innocuous and did not require further intervention to remedy.

## Appendix

### Health update questionnaire

Name: ________________________ Date: _____________

There are events and situations that occur in our lives that can influence our health and well-being. When running a clinical trial it is important for researchers to be aware of any events or situations that may influence the findings of the trial.

This questionnaire is designed to identify any factors that may influence the findings of the trial, and hence influence the final interpretation of results.

We ask that you fill out the questionnaire as accurately as possible. This information, when combined with the data obtained from initial and subsequent assessments, will help researchers accurately interpret the findings at the end of the trial.

Q1) Have you changed your medication or changed the way in which you are managing your condition (Hypothyroidism) in the last 6 months since beginning the trial? (Please circle one)

Yes No

If so, what have you changed?

________________________________________________________________________________________________

Q2) Have you tried any other forms of treatment for your condition in the last 6 months since beginning the trial? (Please circle one)

Yes No

If so, what have you tried?

________________________________________________________________________________________________

Q3) Have there been any major stressful situations or events in the last 6 months that may have influenced you general health and well-being? (Please circle one)

Yes No

If so, what were these situations or events? (Optional)

________________________________________________________________________________________________

Q4) Have you made any significant alterations to your life or your lifestyle in the last 6 months since beginning the trial? (Please circle one)

Yes No

If so, what alterations have you made? (Optional)

________________________________________________________________________________________________

Thank you for taking the time to fill out this questionnaire

## Abbreviations

DASS-42: Depression anxiety stress scale-42 item
DQ: Demographic questionnaire
FT4: Free-Thyroxine
HPA: Hypothalamic-pituitary-adrenal
HUQ: Health update questionnaire
LT4: Levothyroxine
QOL: Quality of life
RCT: Randomized control trial
RHR: Resting Heart Rate
RT: Resting Temperature
SF-36v2: Short form 36 version 2
TFT: Thyroid function test
Tg-Ab: Thyroglobulin antibody
Tg: Thyroglobulin protein
TPO-Ab: Thyroid peroxidase antibody
TPO: Thyroid peroxidase enzyme
TSH: Thyroid stimulating hormone
